# Do Gender and Age Moderate the Relationship between Friendship Quality and Non-Suicidal Self-Injury in Community Children and Adolescents?

**DOI:** 10.5334/pb.1067

**Published:** 2021-11-11

**Authors:** Kristina Eggermont, Margot Bastin, Koen Luyckx, Laurence Claes

**Affiliations:** 1 Faculty of Psychology and Educational Sciences, KU Leuven, Leuven, Belgium; 2 UNIBS, University of the Free State, Bloemfontein, South Africa; 3 Faculty of Medicine and Health Sciences (CAPRI), University of Antwerp, Antwerp, Belgium

**Keywords:** non-suicidal self-injury, friendship quality, adolescence, gender, moderation

## Abstract

In the present study, we investigated the relationship between friendship quality (dimensions) and non-suicidal self-injury (NSSI) as well as the moderating role of gender and age in this relationship. The sample consisted of 463 children and adolescents (50.10% female, age range: 9–17 years). Friendship quality and NSSI were measured using the Friendship Qualities Scale (FQS; Bukowski, Hoza, & Boivin, 1994) and the Self Harm Inventory (SHI; Sansone, Wiederman, & Sansone, 1998), respectively. Overall, total friendship quality and NSSI were significantly and negatively related. Additionally, the relationship between total friendship quality and NSSI was moderated by gender and age. Specifically, girls with low friendship quality reported more NSSI; whereas for boys an opposite effect was found. As for age, friendship quality and NSSI were positively related in older participants. In younger participants, a relationship between friendship quality and NSSI seemed rather absent. This study highlights the important association between friendship quality and NSSI, as well as gender- and age-related differences in this association, which should be taken into account in the prevention and treatment of NSSI.

## Introduction

### Non-suicidal Self-injury

Non-suicidal self-injury (NSSI) is defined as socially unaccepted, direct, and deliberate destruction of one’s own body tissue without suicidal intent, such as cutting, burning, hitting, and scratching oneself (Giletta et al., 2012). NSSI is often associated with psychiatric disorders, the borderline personality disorder (Jacobson & Gould, 2007) and suicide attempts (Nock et al., 2006). The pooled lifetime prevalence of NSSI is estimated around 17.2% for community adolescents (Swannell et al., 2014), and even higher in adolescent inpatients (12–82.4%; Heath et al., 2008). The age of onset of NSSI is situated around 12 to 14 years (Jacobson & Gould, 2007) and NSSI has been found to increase in adolescence and then decrease in young adulthood (Plener et al., 2015). Some studies have reported that girls are more likely than boys to engage in NSSI (Jacobson & Gould, 2007); whereas other studies have not found gender differences (Swannell et al., 2014). However, clear gender differences exist in the applied methods of NSSI: boys are more likely to hit themselves, whereas girls are more likely to cut themselves (Baetens et al., 2011; Heath et al., 2008).

### Peer Relations and NSSI

In adolescence, peer relationships increase in importance as compared to relationships with parents (Brown & Larson, 2009; Rubin et al., 2008). Research has shown that peer support is positively associated with mental well-being and protects adolescents against depression, anxiety, stress, and suicide (Gorrese, 2016; Roach, 2018). However, problems in peer relationships can have a negative impact on adjustment and well-being (Gorrese, 2016; Moore et al., 2017; Tsaousis, 2016; van Geel, Vedder, & Tanilon, 2014).

Several studies have already demonstrated an association between peer relationship characteristics and NSSI (Grigoryan & Jurcik, 2020). A meta-analysis of van Geel, Goemans, and Vedder (2015) demonstrated that peer victimization was positively related to NSSI in children and adolescents. Also, in a cross-sectional study of Gandhi et al. (2016), peer alienation was positively associated with NSSI in high school students. Esposito, Bacchini, and Affuso (2019) found that peer rejection augmented the risk of NSSI engagement in adolescents. Furthermore, in a retrospective study by Heath et al. (2009), university students who engaged in NSSI reported less social support from peers than students who did not engage in NSSI. Giletta et al. (2015) followed adolescents over a period of two years’ time and found that adolescents with low frequency of NSSI engagement reported more friend support than adolescents with higher frequency of NSSI engagement. Finally, adolescent girls who reported more negative perceptions of peers (e.g. “Other kids will try to put you down or tease you if they have a chance”) were found to be more at risk for NSSI onset in the following year (Victor et al., 2019). Hence, given that peer relations increase in importance in adolescence and contribute to psychological well-being/ill-being, peer relations could play an important role in NSSI engagement in adolescents.

## Friendship Quality and NSSI

Bukowski, Hoza, and Boivin (1994) define friendship quality as a concept consisting of five separate dimensions: (1) companionship represents the amount of voluntary time two friends spend together, (2) help indicates how much mutual help persons experience in their friendship, (3) security implies the belief of being able to trust and rely upon a friend, (4) closeness represents the strength of a friendship bond that is experienced between two friends, and (5) conflict is the amount of annoyance, arguments, and fights persons have with their friend. Conflict is the only negative component of the five friendship quality dimensions as conceptualized by Bukowski, Hoza, and Boivin (1994). A substantial amount of research has found associations between peer relationships and NSSI. However, research examining the relation between NSSI and friendship quality dimensions as conceptualized by Bukowski, Hoza, and Boivin (1994) is scarce. Investigating these specific associations could be of added value to the literature since the conceptualization of friendship quality as a multi-dimensional concept (with both positive and negative aspects to it) allows for a more nuanced examination of the link between friendship quality and NSSI.

### The Role of Gender and Age in the Relationship between Friendship Quality and NSSI

Regarding gender differences with respect to friendship quality dimensions, studies have shown that girls score significantly higher on companionship, help, security, and closeness than boys (Bastin et al., 2018; Linden-Andersen, Markiewicz, & Doyle, 2009); whereas girls score lower (Bastin et al., 2018) or similar (Rose & Rudolph, 2006) on conflict compared to boys. With regard to the meaning of friendship, girls are more connection-oriented in their friendships than boys (Rose & Rudolph, 2006). They care more than boys about having a dyadic friendship and worry more about the loss of it (Henrich et al., 2001). Several studies have shown moderating effects of gender in the relationship between friendship/peer relationship characteristics and internalizing symptomatology, in which this association was stronger for girls than boys (Gorrese, 2016; Oldenburg & Kerns, 1997). More specifically, for girls, the support of a friend/classmate negatively predicted internalizing symptoms (i.e., depressive symptoms); whereas for boys, no such association was found (Attar-Schwartz, Mishna, & Khoury-Kassabri, 2019; Slavin & Rainer, 1990). Furthermore, Schmidt and Bagwell (2007) reported that higher levels of security in a friendship acted as a buffer against depressive symptoms in girls but not in boys. These findings imply that there may exist gender differences in the relationship between friendship quality and mental health problems (such as NSSI).

With respect to age, research has shown that friendship quality increases from middle childhood to adolescence and throughout adolescence (Sánchez-Queija & Oliva, 2015; Way & Greene, 2006; Xu, Eggum-Wilkens, & Bradley, 2020): loyalty, intimacy and self-disclosure seem to increase during adolescence (Buhrmester, 1990; Rubin et al., 2008; Weiss & Smith, 2002). Furthermore, Rubin et al. (2008) reported that friendships at age 7 to 8 focus on convenience and rewards/costs; whereas friendships at age 10 to 11 focus on loyalty and sharing the same values. At age 11 to 13, active attempts are made to understand each other and to disclose oneself. In adolescence, the notion of possessiveness is less present in friendship relations and adolescents are more aware of the importance of close relationships for their personal growth (Rubin et al., 2008). To our knowledge, research on age differences in the relationship between peer relationships/friendship quality and NSSI is limited. Yet, as the meaning of friendship changes during adolescence, and as friendship quality tends to increase, its relationship to psychological well-/ill-being (and more specifically NSSI) may evolve as well. For example, as older adolescents disclose more of their feelings and thoughts to their friends and as friends support each other more in their personal growth, friendship in older adolescents could serve as a buffer against NSSI engagement due to increased social support. On the other hand, as intimacy and self-disclosure increase during adolescence, sharing experiences of NSSI engagement with a friend may increase as well, making older adolescents more vulnerable to NSSI socialization (as we know that exposure to peer NSSI can be a risk factor for NSSI, e.g. Fox et al., 2015; Grigoryan & Jurcik, 2020).

### Present Study

In the present study, we investigated the relationship between friendship quality dimensions and NSSI in a community sample of children and adolescents. Based on previous studies (Gandhi et al., 2016; Heath et al., 2009; van Geel, Goemans, & Vedder, 2015), we expected a negative relation between total friendship quality and NSSI. Regarding the friendship quality dimensions, we hypothesized negative relations between the four positive dimensions of friendship quality and NSSI, and a positive association between conflict and NSSI. Furthermore, we examined the role of gender and age as moderators in the relation between friendship quality (dimensions) and NSSI. Based on research on gender differences in the association between friendship quality and psychopathology, we hypothesized that the association between friendship quality and NSSI would be more pronounced in girls compared to boys. Furthermore, as the meaning of friendship changes throughout childhood and adolescence (Buhrmester, 1990; Rubin et al., 2008; Way & Greene, 2006; Weiss & Smith, 2002), we explored the moderation effect of age in the relation between friendship quality (dimensions) and NSSI.

## Method

### Participants & Procedure

Thirteen primary and secondary schools in the Dutch speaking part of Belgium were approached by six master students in psychology in the context of their master’s thesis to partake in the study. These students selected schools based on geographical convenience (e.g., schools in their hometown). Eleven out of the thirteen addressed schools agreed to partake in the study. Each master student was responsible for the data collection in a few classes in a particular school. In total, 504 students were invited to participate in the study. The participants received an informed consent letter which they had to hand over to their parents, in which the purpose of the study was explained. Twenty-six students did not receive parental consent and were not allowed to participate in the study. Before the start of the study, the children and adolescents were invited to read the informed consent letter and to indicate whether they assented to participate. Participants filled out the questionnaires in their class room during school hours. In each class, a master student was present to address questions or emotional distress. At the end of the questionnaire booklet, information was provided on services participants could contact in case of emotional distress or in case they wanted to discuss their symptoms after completion of the questionnaires. They were also advised to contact their general practitioner in case of severe distress. Afterwards, participants were rewarded with sweets. This study was part of a larger study consisting of three measurement waves, but focused on data collected at the second wave given that NSSI was only measured from the second wave onwards. Fifteen students were absent at the second wave. These students did not significantly differ from the final sample with regard to age, *t*(13.46) = 1.25, *p* > .05, or gender, *F*(1,475) = 0.27, *p* > .05.

The final sample (participants who received parental consent and were present at the second wave) consisted of 463 participants of whom 49.90% were boys (*n* = 231), and 50.10% were girls (*n* = 232). The mean age of the sample was 12.86 years (*SD* = 2.09, *range* = 9–17 years). About 415 adolescents (89.60%) in this sample had the Belgian nationality; 6.90% (*n* = 32) had the Dutch nationality, and 3.50% (*n* = 16) of the participants had other nationalities. Almost 22% (21.80%, *n* = 101) of the adolescents were in the fifth, 18.60% (*n* = 86) in the sixth; 8.40% (*n* = 39) in the seventh; 9.70 % (*n* = 45) in the eighth; 16.20% (*n* = 75) in the ninth, and 25.30% (*n* = 117) in the tenth grade. For our analyses, we divided the sample in two age groups: group 1 (age 9–12, *n* = 215) and group 2 (age 13–17, *n* = 247).

### Measures

To measure the quality of their friendship, participants completed the Dutch version of the Friendship Qualities Scale (FQS; Bukowski, Hoza and Boivin, 1994; Vanhalst, Luyckx & Goosens, 2013). The FQS is a self-report questionnaire which consists of 23 items to measure five dimensions of friendship quality, being help (5 items; e.g., “If other kids were bothering me, my friend would help me”), security (5 items; e.g,. “If my friend or I do something that bothers the other one of us, we can make up easily”), closeness (5 items; e.g., “I feel happy when I am with my friend”), companionship (4 items; e.g., “My friend and I spend all our free time together”), and conflict (4 items; e.g,. “I can get into fights with my friend”). Items are rated on a 5-point Likert scale ranging from 1 ‘does not apply to me at all’ to 5 ‘applies to me very well’. Participants were asked to fill in the questionnaire with their same sex best friend in mind. Internal consistency of the total friendship quality scale was excellent with a Cronbach’s alpha coefficient of .88. Internal consistency of the five subscales were as follows: help (α = .82), security (α = .69), closeness (α = .77), companionship (α = .65), and conflict (α = .63).

To assess NSSI, we used the NSSI subscale of the Dutch version of the Self Harm Inventory (SHI; Sansone, Wiederman, & Sansone, 1998). The NSSI subscale assesses five different forms of lifetime NSSI, being self-cutting, burning, hitting, scratching, and head-banging by means of a YES/NO format (Sansone, Songer, & Sellbom, 2006), e.g., “Have you ever deliberately cut yourself?”. Lifetime NSSI was coded as a dichotomous variable, in which a score of 0 means that the participants have never engaged in NSSI during their lifetime and a score of 1 means that the participants have engaged at least once in NSSI during their lifetime. The Cronbach’s alpha coefficient of the NSSI subscale in the present study was .71.

### Analyses

To examine our first research question, i.e., studying the relation between friendship quality (dimensions) and lifetime NSSI, point biserial correlations were calculated. Correlation analyses were performed for the total group, for girls and boys, and for the two age groups (age 9–12; age 13–17) separately.

To address our second research question, that is, the moderating role of gender and age in the association between friendship quality (dimensions) and lifetime NSSI, hierarchical logistic regression analyses were performed, with friendship quality (dimensions), gender, age and their two-way (friendship quality × gender; friendship quality × age) interactions as independent variables and lifetime NSSI as dependent variable. Before performing regression analyses, we standardized all continuous predictors. Analyses were run separately for the total friendship score and for each of the five friendship quality dimensions separately, given that we were specifically interested in how each dimension was related to NSSI.

## Results

### Preliminary Analyses

#### NSSI frequency as a function of gender and age

About 34.10% (*n* = 158) of all participants reported at least one type of NSSI during their lifetime. No significant gender difference was found in the presence/absence of lifetime NSSI, χ²_(1)_ = 1.55, *p* = .21, φ = –.06. About 31.47% (*n* = 73) of girls and about 36.96% (*n* = 85) of boys engaged in lifetime NSSI. However, a significant age difference was found with regard to lifetime NSSI presence/absence: 44.86% (*n* = 96) of the younger age group reported at least one type of NSSI, compared to 25.10% (*n* = 62) of the older group, χ²(1) = 19.87, *p* < .001, φ = –.21. ***Table 1*** displays the frequencies of the different methods of NSSI (for the total sample, boys/girls, and for both age groups; age 9–12; age 13–17). With respect to NSSI methods, boys reported significantly more scratching than girls; whereas younger participants reported significantly more self-beating, head-banging and scratching compared to older participants.

**Table 1:** Frequencies of five NSSI behaviors for the total sample and in function of gender and age.


	TOTAL		GIRLS		BOYS			AGE GROUP 1 (AGE 9–12)		AGE GROUP 2 (AGE 13–17)		

	%	*N*	%	*N*	%	*N*	χ² (φ)	%	*N*	%	*N*	χ² (φ)

Cutting	8.90	41	9.48	22	8.22	19	0.23 (.02)	8.37	18	9.31	23	0.12 (.02)

Burning	3.50	16	2.59	6	4.33	10	1.05 (–.05)	5.12	11	2.02	5	3.25 (–.08)

Beating	19	88	17.24	40	20.78	48	0.94 (–.04)	30.70	66	8.91	22	35.40*** (–.28)

Head-banging	21.60	100	18.53	43	24.78	57	2.66 (–.08)	30.37	65	14.17	35	17.72*** (–.20)

Scratching	16.20	75	12.50	29	19.91	46	4.69* (–.10)	22.32	48	10.93	27	10.97** (–.15)


*Note*: * *p* < .05, ** *p* < .01, *** *p* < .001.

#### Friendship quality (dimensions) as a function of gender and age

Means and standard deviations of friendship quality dimensions are presented in ***Table 2***. For all friendship quality dimensions, except for conflict, girls scored significantly higher than boys. For age, older participants scored significantly higher than the younger ones, except for the subscales closeness and conflict.

**Table 2:** Means, standard deviations and ranges of all friendship quality variables for the total sample, and in function of gender (controlled for age) and age (controlled for gender).


	TOTAL	GIRLS	BOYS	*F* (*η_p_^2^*)	AGE GROUP 1 (AGE 9–12)	AGE GROUP 2 (AGE 13–17)	*F* (*η_p_^2^*)	RANGE
	
	*M (SD)*	*M (SD)*	*M (SD)*		*M (SD)*	*M (SD)*		

Total FQ	89.40 (13.16)	93.98 (11.36)	84.71 (13.24)	64.30*** (.13)	87.16 (14.42)	91.38 (11.61)	13.97*** (.03)	43–113

Companionship	14.11 (3.10)	14.77 (2.87)	13.43 (3.19)	22.31*** (.05)	13.57 (3.36)	14.58 (2.78)	12.73*** (.03)	4–20

Conflict	8.38 (3.02)	8.21 (2.94)	8.55 (3.10)	1.55 (.004)	8.56 (3.23)	8.23 (2.82)	1.33 (.003)	4–20

Help	20.22 (3.95)	21.32 (3.45)	19.10 (4.13)	39.23*** (.08)	19.33 (4.42)	21 (3.31)	23.16*** (.05)	5–25

Security	19.74 (3.75)	21.17 (3.14)	18.28 (3.76)	80.37*** (.15)	19.20 (3.95)	20.23 (3.50)	10.61** (.02)	9–25

Closeness	19.72 (3.72)	20.94 (3.03)	18.48 (3.94)	53.73 *** (.11)	19.63 (3.98)	19.80 (3.47)	0.34 (.001)	5–25


*Note*: Total FQ = Total score of the Friendship Qualities Scale (FQS). * *p* < .05, ** *p* < .01, *** *p* < .001.

### Correlations between Friendship Quality (Dimensions) and NSSI

Point biserial correlation analyses were performed to investigate the associations between friendship quality (dimensions) and lifetime NSSI in the total sample, for girls and boys and for the two age groups separately (see ***Table 3***). Total friendship quality was negatively correlated with lifetime NSSI. With regard to positive friendship quality dimensions, help and security were negatively correlated with lifetime NSSI, whereas companionship and closeness did not show significant correlations with lifetime NSSI. Finally, conflict was positively associated with lifetime NSSI.

**Table 3:** Correlations between the (dimensions of) friendship quality and lifetime NSSI.


	LIFETIME NSSI				

	TOTAL	GIRLS	BOYS	AGE GROUP 1 (AGE 9–12)	AGE GROUP 2 (AGE 13–17)

Total FQ	–.11*	–.24***	.03	–.23**	.11

Companionship	–.04	–.06	.003	–.09	.10

Conflict	.14**	.22**	.06	.10	.18**

Help	–.10*	–.21**	.005	–.23**	.17**

Security	–.13**	–.26***	–.001	–.27**	.08

Closeness	0	–.12	.13	–.15*	.19**


*Note*: Total FQ = Total score of the Friendship Qualities Scale (FQS). * *p* < .05, ** *p* < .01, *** *p* < .001.

For girls, total friendship quality was found to be negatively correlated with lifetime NSSI. Furthermore, for this group, help and security were significantly negatively correlated with lifetime NSSI and conflict was significantly positively correlated with lifetime NSSI. For boys however, no significant associations were found between total friendship quality and lifetime NSSI.

Regarding age, the younger age group showed a significant negative correlation between total friendship quality and lifetime NSSI, whereas no such correlation was found in the older age group. Furthermore, help, security and closeness were significantly negatively correlated with lifetime NSSI in the younger age group; whereas help and closeness were significantly positively correlated with lifetime NSSI in the older age group. Finally, conflict was positively correlated with lifetime NSSI only in the older age group.

### Gender and Age as Moderators in the Relationship Between Friendship Quality and NSSI

***Table 4, 5, 6, 7, 8, 9*** display the results of the regression analyses with lifetime NSSI as independent variable.^1^,^2^

**Table 4:** Prediction of lifetime NSSI based on total friendship quality, gender, age and their interactions.


STEP	VARIABLES	*B*	*WALD’S* *χ²*	*NAGELKERKE* *R²*

Step 1	Total FQ	–0.15	1.80	.07

	Gender	0.16	0.53	

	Age	–0.42	15.81***	

Step 2	Total FQ	0.27	2.72	.12

	Gender	0.23	1.05	

	Age	–0.42	14.39***	

	Total FQ × gender	–0.63	7.17**	

	Total FQ × age	0.37	9.73**	


*Note*: Total FQ = Total score of the Friendship Qualities Scale (FQS). * *p* < .05, ** *p* < .01, *** *p* < .001.

**Table 5:** Prediction of lifetime NSSI based on companionship, gender, age and their interactions.


STEP	VARIABLES	*B*	*WALD’S* *χ²*	*NAGELKERKE* *R²*

Step 1	Companionship	0.03	0.07	.06

	Gender	0.28	1.74	

	Age	–0.45	17.86***	

Step 2	Companionship	0.16	1.25	.07

	Gender	0.29	1.88	

	Age	–0.47	18.51***	

	Companionship × gender	–0.13	0.39	

	Companionship × age	0.21	3.49	


* *p* < .05, ** *p* < .01, *** *p* < .001.

**Table 6:** Prediction of lifetime NSSI based on conflict, gender, age and their interactions.


STEP	VARIABLES	*B*	*WALD’S* *χ²*	*NAGELKERKE* *R²*

Step 1	Conflict	0.30	8.34	.09

	Gender	0.24	1.39	

	Age	–0.44	18.09***	

Step 2	Conflict	0.17	1.35	.10

	Gender	0.26	1.54	

	Age	–0.45	18.05***	

	Conflict × gender	0.34	2.67	

	Conflict × age	0.10	0.82	


* *p* < .05, ** *p* < .01, *** *p* < .001.

**Table 7:** Prediction of lifetime NSSI based on help, gender, age and their interactions.


STEP	VARIABLES	*B*	*WALD’S* *χ²*	*NAGELKERKE* *R²*

Step 1	Help	–0.10	0.94	.06

	Gender	0.20	0.90	

	Age	–0.42	15.43***	

Step 2	Help	0.32	3.75	.12

	Gender	0.27	1.58	

	Age	–0.47	16.39***	

	Help × gender	–0.51	5.06*	

	Help × age	0.43	12.78***	


* *p* < .05, ** *p* < .01, *** *p* < .001.

**Table 8:** Prediction of lifetime NSSI based on security, gender, age and their interactions.


STEP	VARIABLES	*B*	*WALD’S* *χ²*	*NAGELKERKE* *R²*

Step 1	Security	–0.21	3.49	.07

	Gender	0.10	0.21	

	Age	–0.41	15.38***	

Step 2	Security	0.17	1.16	.13

	Gender	0.12	0.29	

	Age	–0.39	12.90***	

	Security × gender	–0.71	8.23**	

	Security × age	0.37	10.18**	


* *p* < .05, ** *p* < .01, *** *p* < .001.

**Table 9:** Prediction of lifetime NSSI based on closeness, gender, age and their interactions.


STEP	VARIABLES	*B*	*WALD’S* *χ²*	*NAGELKERKE* *R²*

Step 1	Closeness	0.05	0.20	.06

	Gender	0.29	1.80	

	Age	–0.44	17.95***	

Step 2	Closeness	0.45	7.82	.12

	Gender	0.34	2.34	

	Age	–0.46	17.30***	

	Closeness × gender	–0.59	6.12*	

	Closeness × age	0.43	13***	


* *p* < .05, ** *p* < .01, *** *p* < .001.

#### Moderating effect of gender in the relationship between friendship quality and NSSI

**Figure 1:**
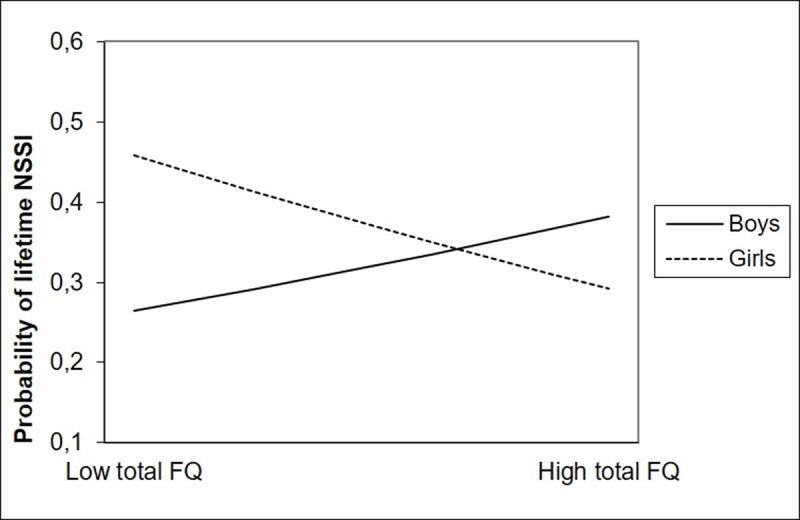
Interaction between total friendship quality and gender in the prediction of lifetime NSSI. *Note*: Total FQ = Total score of the Friendship Qualities Scale (FQS).

**Figure 2:**
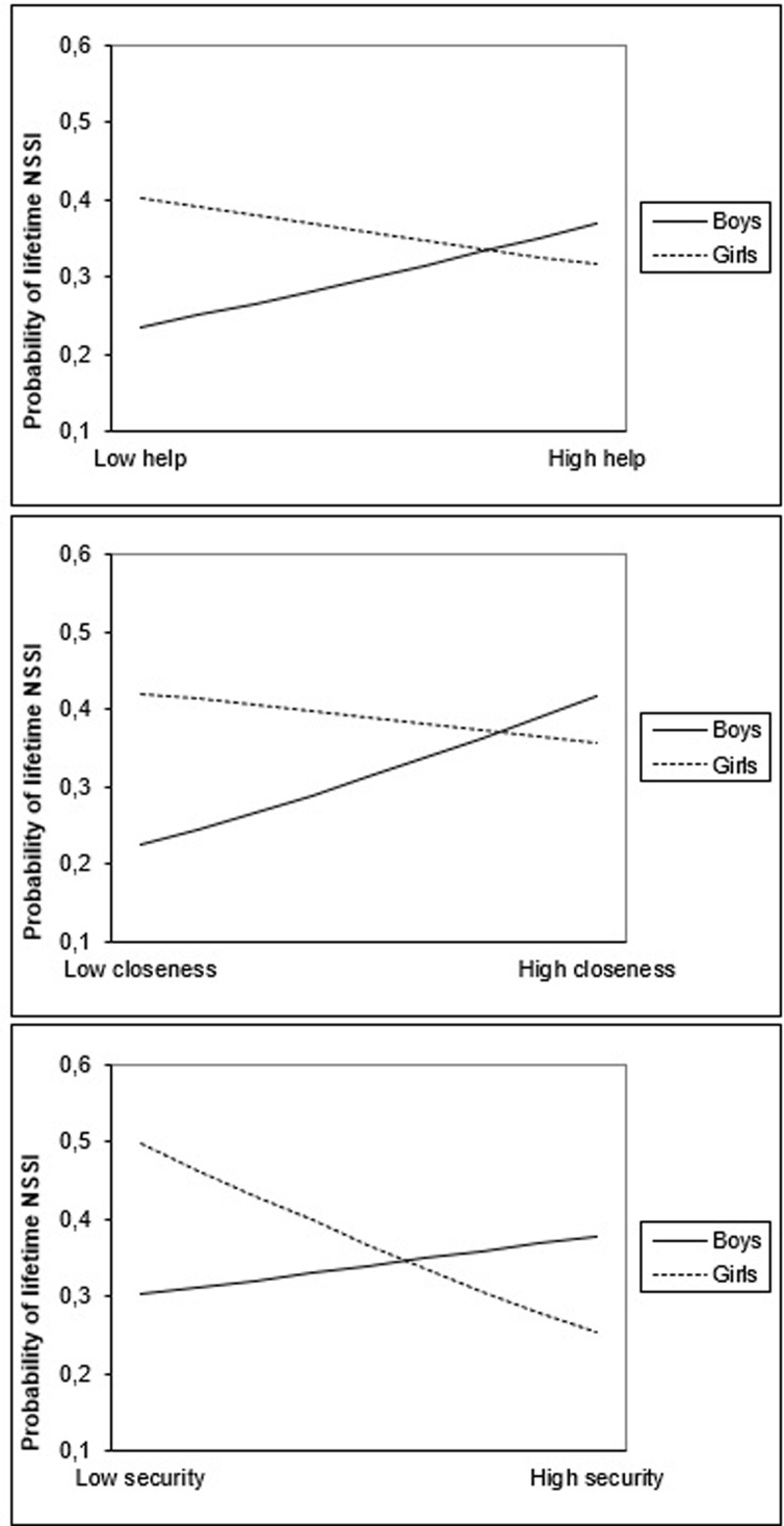
Interaction between help and gender, security and gender, and closeness and gender in the prediction of lifetime NSSI.

For total friendship quality, lifetime NSSI was negatively predicted by the interaction between friendship quality and gender. ***Figure 1*** shows the nature of this interaction effect: for girls, NSSI engagement increased when friendship quality decreased. For boys, the opposite effect was found. Gender differences were most distinct in low friendship quality. With regard to friendship quality dimensions, gender was a significant moderator in the relation between help, closeness, and security and lifetime NSSI. As ***Figure 2*** shows, for girls, NSSI engagement increased when help, closeness, and security decreased, whereas for boys, the opposite was found. No significant interaction between gender and companionship and conflict were found in the prediction of lifetime NSSI.

#### Moderating effect of age in the relationship between friendship quality and NSSI

**Figure 3:**
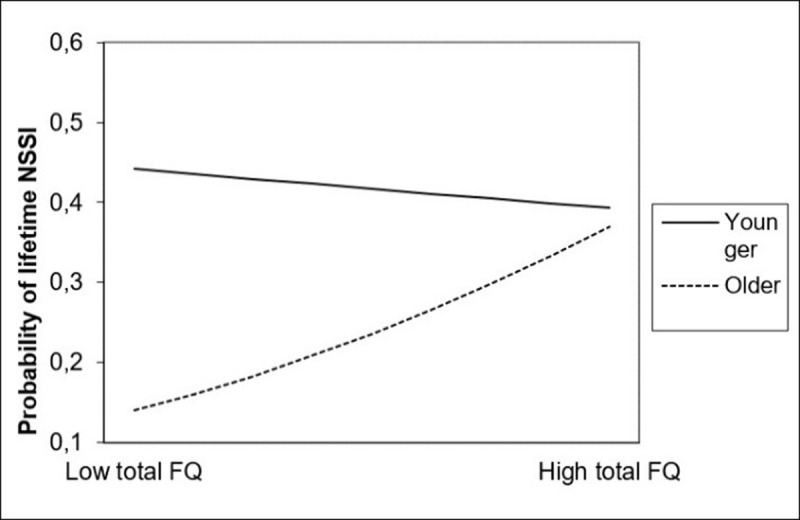
Interaction between total friendship quality and age in the prediction of lifetime NSSI. *Note*: Total FQ = Total score of the Friendship Qualities Scale (FQS). Younger = age at one standard deviation under mean age; Older = age at one standard deviation above mean age.

**Figure 4:**
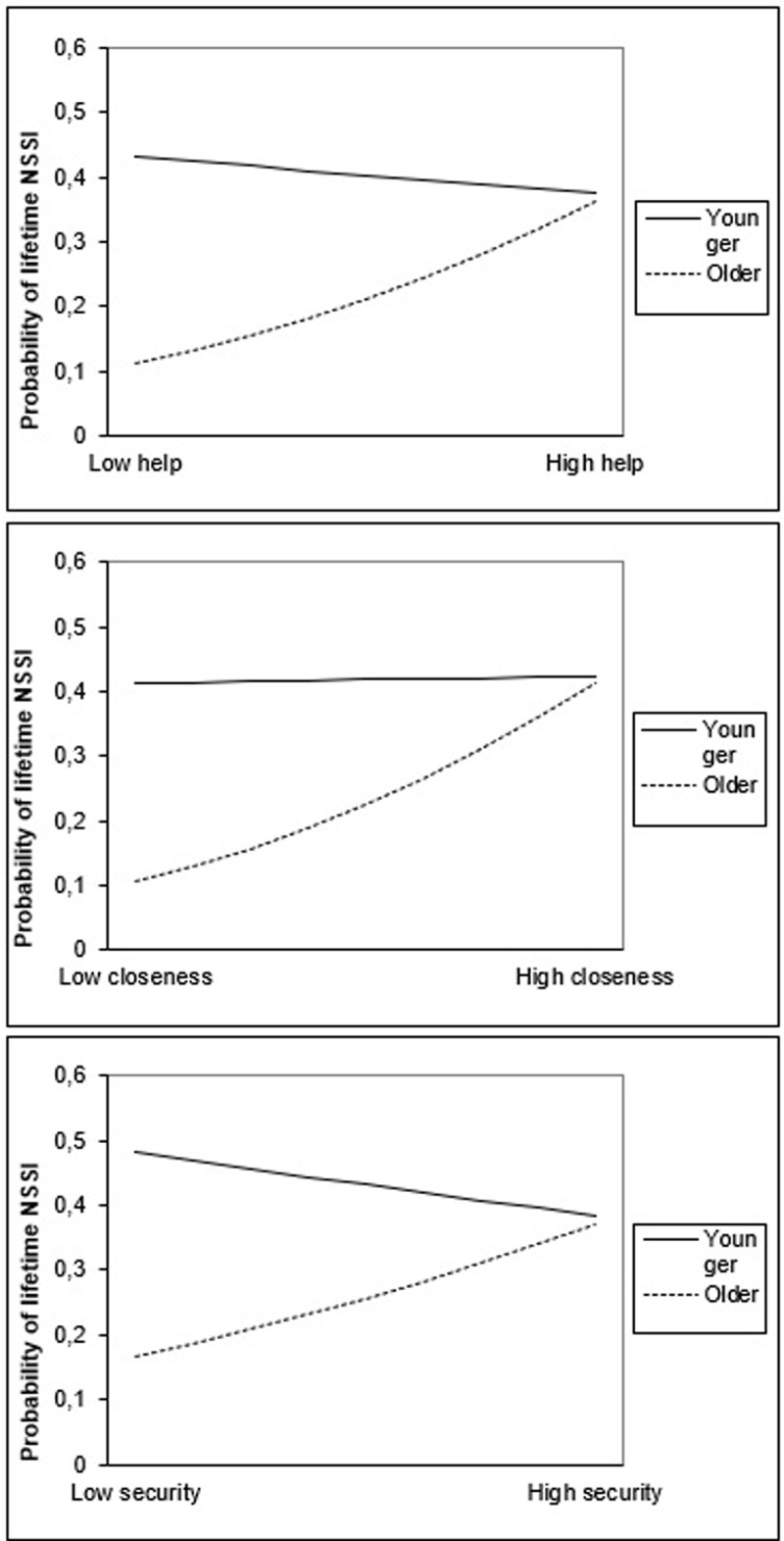
Interactions between help and age, closeness and age, and security and age in the prediction of lifetime NSSI. *Note*: Younger = age at one standard deviation under mean age; Older = age at one standard deviation above mean age.

Age as well turned out to be a moderator in the relationship between total friendship quality and lifetime NSSI. ***Figure 3*** displays the nature of this interaction: for older participants, NSSI engagement increased along with increases in friendship quality. For younger participants, no such relationship was found. Regarding friendship quality dimensions, the interaction between age on the one hand and help, closeness and security on the other hand positively predicted lifetime NSSI. ***Figure 4*** displays the nature of these interaction effects, which is similar to the interaction effect between total friendship quality and age. No significant interactions between age and companionship and between age and conflict were found in the prediction of lifetime NSSI.

## Discussion

This study aimed to investigate the associations between friendship quality (dimensions) and NSSI as well as to examine the moderating role of age and gender in these associations.

About one third of the participants (34.10%) engaged in at least one type of lifetime NSSI. No gender differences were found in the prevalence of lifetime NSSI. However, boys reported significantly more scratching compared to girls. Furthermore, the younger age group (9–12 years) reported significantly more lifetime NSSI than the older age group (13–17 years), and more specific, younger participants reported significantly more self-beating, head-banging, and scratching than older participants. This finding is not in line with previous studies, in which NSSI was found to increase during adolescence (Plener et al., 2015). More research is needed to investigate age-related effects on NSSI behaviors.

With respect to friendship quality, our findings showed that girls scored significantly higher on all positive friendship quality dimensions and total friendship quality compared to boys, comparable to what has been mentioned in previous studies (Bastin et al., 2018; Linden-Andersen, Markiewicz, & Doyle, 2009). In line with Rose and Rudolph (2006), no gender differences were found for the negative friendship quality dimension conflict. With regard to age, our results confirm previous findings that showed that friendship quality increases during adolescence (Buhrmester, 1990; Rubin et al., 2008; Way & Greene, 2006; Weiss & Smith, 2002). Only conflict and closeness did not significantly differ between younger and older participants.

Total friendship quality and lifetime NSSI were significantly and negatively correlated, as expected (Giletta et al., 2015; Heath et al., 2009). Negative correlations were found between the positive friendship quality dimensions help and security and NSSI, whereas conflict was positively associated with NSSI, also confirming our hypotheses.

With regard to gender, no significant associations between friendship quality (dimensions) and NSSI were found for boys. For girls on the contrary, there were significant negative correlations between NSSI and total friendship quality, help, and security. There was a significant positive correlation between conflict and lifetime NSSI for girls. Furthermore, gender moderated the relationship between friendship quality (total and friendship quality dimensions help, closeness, and security) and NSSI. As expected for girls, NSSI engagement increased when friendship quality decreased. For boys, however, the opposite was found. Gender differences were more prominent in low friendship quality (total, help and closeness): when these friendship aspects were low, girls reported higher lifetime NSSI compared to boys. We suggest two possible explanations for this finding. First, as we know from previous research, girls who experience stress tend to seek more support than boys (Rose & Rudolph, 2006). Consequently, girls who have a close friend who provides help and security might be more likely to receive support during stress and they may therefore be able to cope better with stressful situations (as opposed to girls who do not have such high-quality friendships). Second, Henrich et al. (2001) posited that girls tend to worry more about relationship loss. Hence, girls with low quality friendships might be more prone to ruminate about their friendship compared to boys, which could make them more vulnerable to engage in NSSI, as we know that there is a positive relation between rumination and NSSI (Buelens et al., 2019; Hasking et al., 2019; Selby, Connell, & Joiner Jr., 2010). Overall, our results regarding the association between friendship quality and NSSI in boys are not as clear as those in girls, since for example in the correlation analyses, no positive association was found between friendship quality and NSSI in boys. More research is warranted to gain more understanding in the longitudinal directionality and underlying mechanisms in the association between friendship quality and NSSI in girls versus boys.

As for age, in the younger group, total friendship quality, help, closeness, and security were negatively correlated with NSSI. As for the older age group, significant correlations in the opposite direction (i.e. positive correlations) were found between NSSI and the friendship quality dimensions help and closeness. Age also operated as a moderator in the relationship between friendship quality (dimensions help, closeness, and security) and NSSI. For older participants, NSSI engagement increased when total friendship quality, help, closeness, and security increased. For the younger age group, the relationship between these friendship quality dimensions and NSSI engagement seemed rather absent. Further research is needed to understand the nature of these age-related moderation effects between friendship quality and NSSI. As suggested in the introduction, a possible explanation for the positive association between friendship quality and NSSI in older adolescents is an increase in self-disclosure to their friends in this age group and hence a possible increase in exposure to peer NSSI, which in turn could lead to NSSI contagiousness (Fox et al., 2015; Grigoryan & Jurcik, 2020). Overall, our results regarding age-related effects suggest there may be an important developmental factor to the relation between friendship quality and NSSI. However, it should be noted that we do not have any information about the age of onset/occurrence of the reported NSSI behavior(s). In future research, longitudinal (cohort-sequential) designs could be adopted to obtain a more fine-grained investigation of developmental aspects in the relationship between friendship quality and NSSI.

Both in the correlation analyses as in the regression analyses, friendship quality dimension companionship did not show any association with NSSI. A possible explanation lies in the meaning of this friendship quality dimension: companionship concerns merely the amount of time two friends spend together, but does not measure how this time is spent or what the friendship means to a person. This finding suggests that the association between friendship quality and NSSI depends more on the meaning and nature of a friendship (do friends help each other, can they trust and rely upon their friend, do they feel close to their friend) than solely on having a friend to spend time with.

Besides the strengths of our study, some limitations should be addressed. First, the sample in this study consisted of a community sample of boys and girls from fifth to tenth grade. As such, these findings are limited to this age group of community adolescents and cannot be generalized to other age groups and clinical samples. A second limitation lies in the instruments used for this study. All questionnaires were self-report questionnaires and could thus have caused shared method variance. In future research, perceptions of the target’s friend on their relationship could be included as well. Third, this study investigated same-sex friendships only so no conclusions can be drawn regarding friendship quality of opposite-sex friendships. Future research could use a different design than this study whereby reciprocated friendships and opposite-sex friendships would be possible. Fourth, no conclusions on directionality of effects can be drawn based on our results, given the cross-sectional nature of the study. Moreover, the outcome measure in this study was lifetime NSSI, which measures the occurrence of at least one act of NSSI in the respondent’s lifetime; whereas the independent variables of friendship quality refer to the respondent’s current situation. Hence, no conclusions on temporal order between these variables can be made and the findings of this study can only be interpreted as concurrent associations. It would be interesting for future research to adopt a longitudinal study design to examine (bi)directional effects between friendship quality dimensions and NSSI. Furthermore, using ecological momentary assessments in future research could give more insight in the association between friendship quality and NSSI as occurring in very short intervals in daily life.

Some clinical implications can be formulated based on our findings. In general, our study demonstrated significant associations between friendship quality and NSSI engagement in girls. With regard to NSSI prevention, it can be important for therapists and other caretakers to be attentive to peer relations. More specifically, our findings demonstrate that positive friendship quality dimensions (help, closeness, security) are negatively related to NSSI in girls. Hence, it seems important for clinical assessment to not only investigate the presence of friends but also the quality of the friendship. Girls who report low closeness, low help and low security in their friendship may be more vulnerable to engage in NSSI (though again, we cannot draw any causal conclusions from our findings). Girls who are engaging in NSSI could be helped with interventions focusing on interpersonal skills and friendship dynamics. Current prevention and treatment approaches already focus on interpersonal functioning (e.g., Baetens et al., 2020; Gonzales & Bergstrom, 2013; Muehlenkamp, Walsh, & McDade, 2009) but to our knowledge, there do not yet exist prevention or intervention programs specifically targeting friendship quality.

## Footnotes

Linear regression analyses with NSSI versatility (a severity indicator, measured by the sum score of the five NSSI items) as independent variable were performed as well. Since these analyses yielded identical results, we limited ourselves to discussing the results of lifetime NSSI in this section.

Hierarchical regression analyses with three-way interactions, i.e. friendship quality (dimensions) × gender × age, were performed as well. Given that not one three-way interaction was significant, we decided to leave out the discussion of these results in this section. The two-way interaction gender × age that was included in the three-way interaction regression analyses was significant in all regression analyses and indicated the following: older boys reported less lifetime NSSI than younger boys, whereas no clear age differences appeared for girls.
